# From Metabonomics to Pharmacometabonomics: The Role of Metabolic Profiling in Personalized Medicine

**DOI:** 10.3389/fphar.2016.00297

**Published:** 2016-09-08

**Authors:** Jeremy R. Everett

**Affiliations:** Medway Metabonomics Research Group, University of GreenwichKent, UK

**Keywords:** metabonomics, metabolomics, pharmacometabonomics, pharmacometabolomics, personalized medicine, metabolic profiling

## Abstract

Variable patient responses to drugs are a key issue for medicine and for drug discovery and development. Personalized medicine, that is the selection of medicines for subgroups of patients so as to maximize drug efficacy and minimize toxicity, is a key goal of twenty-first century healthcare. Currently, most personalized medicine paradigms rely on clinical judgment based on the patient's history, and on the analysis of the patients' genome to predict drug effects i.e., pharmacogenomics. However, variability in patient responses to drugs is dependent upon many environmental factors to which human genomics is essentially blind. A new paradigm for predicting drug responses based on individual pre-dose metabolite profiles has emerged in the past decade: pharmacometabonomics, which is defined as “the prediction of the outcome (for example, efficacy or toxicity) of a drug or xenobiotic intervention in an individual based on a mathematical model of pre-intervention metabolite signatures.” The new pharmacometabonomics paradigm is complementary to pharmacogenomics but has the advantage of being sensitive to environmental as well as genomic factors. This review will chart the discovery and development of pharmacometabonomics, and provide examples of its current utility and possible future developments.

## Introduction

Many patients experience little or no efficacy, or even suffer toxicity, when prescribed drugs today. A 1998 study by Pomeranz et al. estimated that in US hospitals in 1994, over 2 million patients had serious adverse drug reactions (ADRs) requiring hospitalization, producing permanent disability, or in an estimated 106,000 cases, that led to death (Lazarou et al., 1998). This is a shocking state of affairs given the advances in twenty-first century medicine. It is estimated that the cost to the US economy of ADRs is between $30 and $100 billion per year (Lee et al., 2014).

There is thus a clear need to be able to personalize medicine to ensure that patients are prescribed medications that will be both efficacious and free of noxious side-effects. Personalized medicine has many definitions including “Application of genomic and molecular data to better target the delivery of healthcare, facilitate the discovery and clinical testing of new products, and help determine a person's predisposition to a particular disease or condition” (Abrahams et al., 2005). It is also known as precision medicine, stratified medicine, or individualized medicine. A simpler definition would be “the use of genomic, molecular, and clinical information to select medicines that are more likely to be both effective and safe for that patient” (Everett et al., 2016). Personalized medicine has a long history, as all good physicians and clinicians will tailor their treatments and medication prescription to the needs of the individual patient. However, since the sequencing of the human genome completed, there has been a growing interest in the analysis of human genetic variations, particularly single nucleotide polymorphisms (SNPs), and the correlation of those variations with drug efficacy and safety. There have also been significant developments in the association of genetic variation with differing metabolite profiles or metabotypes (Holmes et al., 2008) between individuals, in genome-wide association studies (GWAS).

Pharmacogenomics is the study of how genetic variation modulates drug responses between individuals and evidence has accumulated of the involvement of over 2000 genes in drug responses (Salari et al., 2012). However, the successful use of pharmacogenomics in the clinic has been limited (Urban and Goldstein, 2014) and recent reviews of the use of pharmacogenomics in randomized clinical trials in cardiovascular disease (Joseph et al., 2014), type-2 diabetes (Maruthur et al., 2014), and depression (Perlis, 2014) have failed to show clear value.

In principle there are several reasons why pharmacogenomics studies on their own may struggle to predict drug responses in humans: (1) drug absorption, metabolism, and excretion will be subject to environmental factors such as diet, the use of alcohol, the taking of other medications, and the status of the patient's microbiome (Holmes et al., 2012); (2) the detection of upstream genetic differences in a patient indicates that there *may* be alterations in the patient's downstream metabolic phenotype, not that there necessarily will be: there is not always a fixed relationship between altered genotype and expression of phenotype, and (3) the issue of phenoconversion, induced by drug co-administration (Shah and Smith, 2015), where a genetic extensive metabolizer can be converted into a phenotypic poor metabolizer and thus confound a pharmacogenomics analysis.

In this situation, the use of metabolic profiling to predict drug efficacy and safety has a number of notable advantages. Firstly, the metabolic phenotype reflects the actual physiological status of the patient in real time, not some future possible state. Secondly, metabolic profiling has the ability to be sensitive to both genetic and environmental factors, including the status of the gut microbiome, that are critical for phenotype expression.

Metabolic profiling of biological fluids, tissues and other samples using various technologies has a history that goes back at least several decades (Lindon and Wilson, 2016). The use of these approaches increased significantly in the 1980s with the advent of advanced pulsed Fourier transform nuclear magnetic resonance (NMR) spectroscopy (Lindon et al., 1999) and hyphenated mass spectrometry (MS) (Theodoridis et al., 2011) analytical technologies capable of profiling dozens to hundreds of metabolites in biological fluids such as urine or blood plasma. Early applications were established in the investigation of drug metabolism (Everett et al., 1984), toxicology (Holmes et al., 1992), inborn errors of metabolism (Iles et al., 1985) and the understanding of disease states (Bales et al., 1984). Metabolic profiling is now termed metabonomics or metabolomics (Lindon et al., 2007).

Metabonomics^1^ has the following *interventional* definition: “the quantitative measurement of the multiparametric metabolic response of living systems to pathophysiological stimuli or genetic modification” (Lindon et al., 2000). The alternative term metabolomics was coined by Fiehn et al. (Fiehn, 2002) and given the following *observational* definition: “a comprehensive analysis in which all the metabolites of a biological system are identified and quantified.” The latter definition is potentially less useful due to both its observational nature and the near impossibility of identifying, let alone quantifying, all the metabolites in a complex biological system. Originally the terms were distinct with metabonomics being used for studies of biofluids and tissues, particularly using NMR detection methodologies, and metabolomics being used for studies of plant and cellular metabolites, particularly by MS. The two terms are nowadays used inter-changeably: we will use the original term metabonomics throughout.

The two main technologies used for metabolic profiling studies are NMR spectroscopy and MS, the latter usually in a hyphenated mode with a separation technology such as gas chromatography (GC), high performance liquid chromatography (HPLC), or ultra performance liquid chromatography (UPLC). The key features of these technologies are briefly summarized in Boxes 1, 2 and the interested reader is referred to consult further literature (Lindon et al., 1999, 2007; Theodoridis et al., 2011; Dona et al., 2016).

Box 1 **NMR spectroscopy**

Nuclear magnetic resonance (NMR) spectroscopy is the most powerful method for the elucidation of the structure of small molecules in solution and it has an important role in the detection, identification, and quantification of metabolites in biological samples, especially in biological fluids. In a typical one-dimensional NMR experiment a sample of a biofluid in a glass tube would be inserted into a probe placed in a strong [usually 14.1 Tesla (T) or above] and highly homogeneous magnetic field that would induce an alignment of NMR-active nuclei with the magnetic field direction. A short (typically microseconds duration) radiofrequency pulse of the correct power and frequency for a given NMR-active nucleus is then applied to the sample which causes the NMR-active nuclei to move out of alignment with the magnetic field. The relaxation of these excited nuclei back to their ground state induces an oscillating electric current in the receiver coils of the probe of the spectrometer that decays, typically over a few seconds. Fourier transformation of this time-domain free induction decay signal gives rise to the familiar frequency-domain NMR spectrum in which signal intensity is plotted as a function of nuclear resonance frequency. The most commonly studied NMR-active nucleus is the proton, ^1^H, which is the most sensitive non-radioactive nucleus and is the workhorse of metabonomics studies. Through the use of a reference standard, typically 3-(trimethylsilyl)-2,2′,3,3′-tetradeuteropropionic acid (TSP) or deuterated forms of 4,4-dimethyl-4-silapentane-1-sulfonic acid (DSS) or its sodium salt, the NMR frequencies of the protons / hydrogen atoms are converted into a dimensionless chemical shift measured in parts per million relative to the reference. This chemical shift is constant, no matter what the magnetic field strength.

The ^1^H NMR spectrum of a biofluid has some remarkable properties:

1. Each chemically non-equivalent hydrogen atom in a metabolite will resonate at its own individual chemical shift which is determined by the chemical nature of that hydrogen and its neighboring atoms in the metabolite and by the solution environment of the sample.
2. Each individual hydrogen atom in a metabolite gives rise to a signal that has an area proportional to the relative concentration of that metabolite in the sample i.e., given certain provisos, ^1^H NMR spectroscopy is a fully quantitative technology and the signal area for a CH_2_ group in a metabolite will be exactly double that of the signal area for a CH moiety in that same metabolite. NMR spectroscopy is thus an excellent, quantitative, non-selective detector, and the ^1^H NMR spectrum of a biofluid will generally equate to the sum of the NMR spectra of all the component metabolites, in proportion to their concentrations in the sample.
3. The nuclei of neighboring, non-equivalent hydrogen atoms in a metabolite will spin-couple to one another, giving rise to ^1^H NMR signal splittings (also known as J-couplings) that are critical for metabolite identification and structure elucidation. These signal splittings or multiplicities obey an *n* + 1 rule in first order spectra, where *n* = the number of equivalent neighboring protons. For instance in the ^1^H NMR spectrum of lactic acid, CH_3_-CH(OH)-CO_2_H, the CH_3_- methyl group protons will resonate as a 2-line, 1:1 intensity doublet (1 + 1 = 2) at 1.33 ppm due to it having one neighboring hydrogen on the adjacent CH group. By contrast the CH proton will resonate as a 4-line, 1:3:3:1 intensity quartet (3 + 1 = 4) as it has three equivalent hydrogen neighbors. The intensity patterns obey Pascal's triangle. The size of the splittings is also informative and depends on the distance in bonds between the coupling hydrogens and their chemical, stereochemical, and conformational properties. Typically, 2-bond couplings, ^2^J_H, H_ between non-equivalent hydrogens on the same carbon atom are larger in size than 3-bond couplings, ^3^J_H, H_, between hydrogens on adjacent carbons, and coupling over 4-bonds or more are generally much smaller.

Many nuclei including ^12^C and ^16^O are NMR-inactive and this may appear to be a disadvantage but in fact, this helps simplify the NMR spectra of metabolites, which would otherwise be hugely complex and difficult to interpret. In metabolite identification, use is made however of the fact that the 1.1% natural abundance ^13^C isotope is NMR-active, and although insensitive to direct detection, can be readily observed via indirect detection through the ^1^H nucleus. This is important as the chemical shift range in ^13^C NMR is 20 times that of ^1^H NMR and thus much more sensitive to subtle changes in metabolite structure. Two-dimensional NMR experiments (see below) that correlate proton chemical shifts with those of directly bonded or more remote carbons are particularly important in metabolite identification.

Huge advances have been made in NMR spectroscopy in the past several decades due to the development of:

1. Higher magnetic fields enabling greater sensitivity and higher signal dispersion.
2. 2-dimensional NMR spectroscopy enabling the spreading out of the NMR spectra of complex samples such as biofluids into a second frequency dimension and the automated correlation of through-bond or even through-space connectivities between NMR-active nuclei, critical to metabolite identification.
3. Cryo-cooled probes giving much higher spectral sensitivity due to the reduction in thermal noise.
4. Automated and highly stable, digital NMR spectrometers giving unmatched reproducibility, quality, and throughput.

The Achilles heel of NMR spectroscopy has been and still is low sensitivity, due to the fact that the signal in NMR comes only from the tiny fraction of nuclei that are in excess in the nuclear magnetic ground state at spin equilibrium. In general the detection limits are in the range mM to uM whereas MS-based techniques like LC-MS can detect metabolites in the range mM down to pM.

Box 2 **Mass spectrometry (MS)**

MS is used as the other main detection technology for metabolic profiling experiments. Mass spectrometers measure the mass-to-charge ratios of charged molecular ions or molecular ion fragments following their ionization in an ion source. In general, MS detection follows a hyphenated, on-line separation step such as GC, HPLC, or UPLC, so that fewer components are introduced into the mass spectrometer at the same time, reducing ionization suppression effects.

After separation by HPLC or UPLC, metabolites are ionized, typically using an ionization technology such as electrospray and often utilizing both positive and negative ion generation and detection. Electrospray ionization (ESI) is a soft ionization method that results in few fragment ions. In the positive ionization mode, ESI+, it will usually give protonated molecular ions [M + H]^+^, or salt or solvent adducts thereof as the most intense ions in the spectrum. In cases where metabolites are unstable, for instance exhibiting a tendency to dehydrate, it is common to observe protonated, dehydrated molecular ion fragments, [M + H − H_2_O]^+^. Correspondingly in negative ion mode the ES- spectrum will consist largely of deprotonated molecular ions or ion complexes.

Separation of metabolites by GC will generally require derivatisation of the metabolites in order to make them volatile enough to travel through the GC column. This can introduce issues of variable metabolite derivatisation both in terms of the efficiency of derivatisation between different metabolites and the possibility that some individual metabolites may exhibit mono-, di-, or poly-derivatisation, resulting in multiple molecular species and spectral interpretation complexity. In GC-MS mode, electron impact ionization is often used, which is a hard ionization technique generating a much greater degree of fragmentation in the mass spectrum and enabling the use of MS libraries for metabolite identification.

The hyphenated mass spectra of a biofluid sample will have the following characteristics:

1. Elution of metabolites from the separation technology at characteristic retention times, allowing confirmation of metabolite identity based on chromatographic retention time, assuming that authentic reference standards of the metabolite are available.
2. Presence of molecular ion, molecular ion adduct or fragment ions that can be characteristic for the metabolite giving rise to them, in terms of mass-to-charge ratios.
3. If experiments are conducted under conditions of high resolution, molecular formula information can be derived on the molecular ions and fragments.
4. Quantitation of a metabolite can be challenging unless a reference standard of the metabolite is available.
5. Sensitivity of metabolite detection is very high: mM down to pM and much superior to NMR spectroscopy.
6. Run-to-run reproducibility is lower than for NMR spectroscopy, due to direct introduction of samples into the spectrometer and the variability that that generates in the spectrometer, particularly in ionization efficiency.

MS is thus highly complementary to NMR spectroscopy and the two technologies are often most powerful when used in concert. Although MS is not a universal detector like NMR spectroscopy, its high sensitivity is a key factor that dictates it choice in many metabolic profiling experiments.

**Figure 1:**
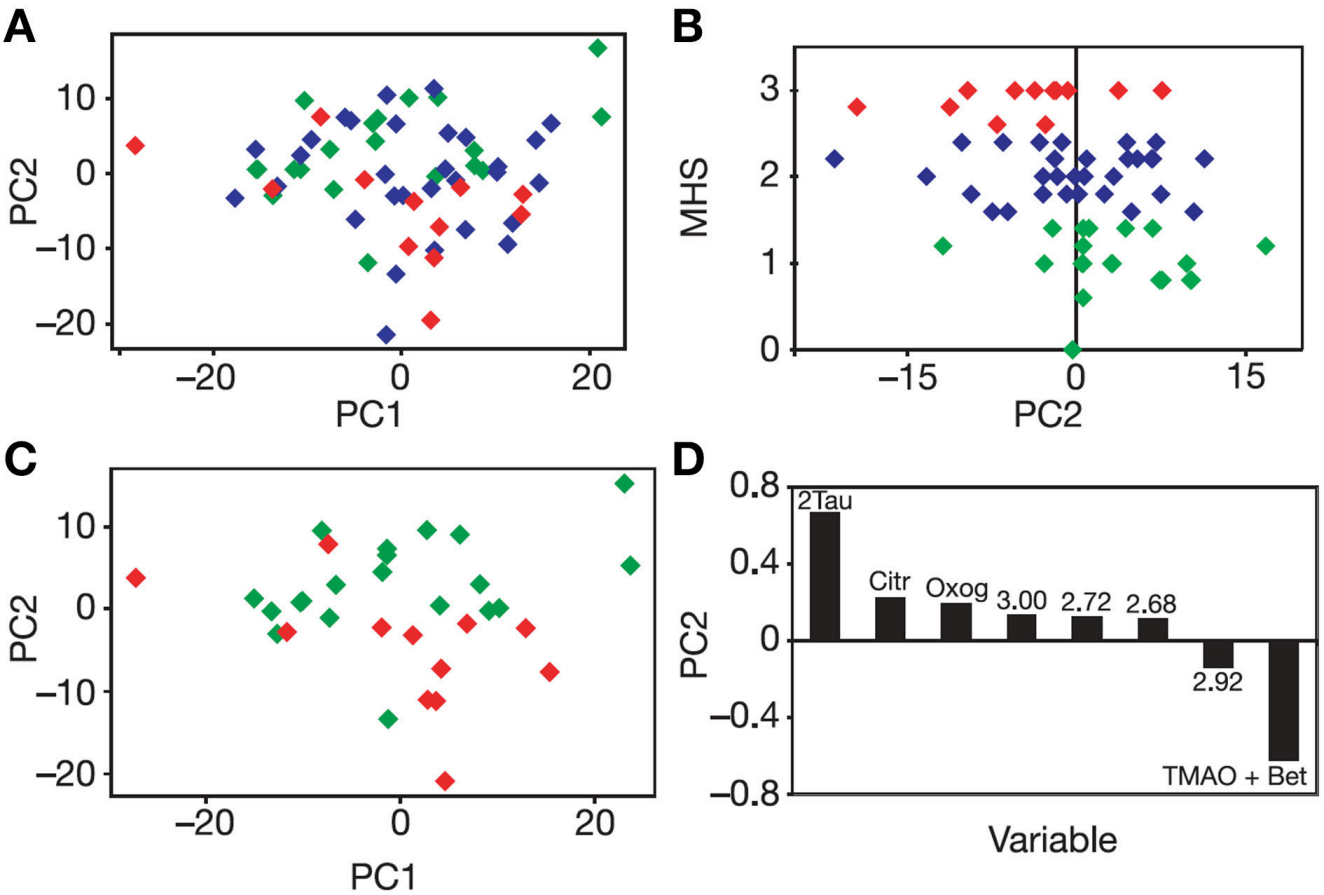
**Unsupervised PCA of the *pre-dose* urines of rats dosed subsequently with paracetamol. (A)** PCA Scores plot of the multivariate analysis of the binned 600 MHz ^1^H NMR spectra of the pre-dose rat urine. Each diamond corresponds to a different animal color-coded according to its mean, post-dose liver histopathology score (MHS): Class 1, no or minimal liver necrosis, green; Class 2, mild necrosis, blue, and Class 3, moderate necrosis, red. A partial separation is observed across PC2 between Class 1 and Class 3. **(B)** A plot of MHS against PC2: a weak but significant correlation is observed. **(C)** PCA Scores plot for the ^1^H NMR spectra of the pre-dose rat urine, with the same color-coding as in **(A)** for Classes 1 and 3 only: the partial separation across PC2 is more readily observed. **(D)** The PCA loadings plot showing the bins of the ^1^H NMR spectrum of the pre-dose urine that are responsible for the separations across PC2 and the direction of influence. Tau, taurine; Citr, citrate; Oxog, 2-ketoglutarate; TMAO + Bet, trimethylamine-*N*-oxide (TMAO) and betaine. Numbers correspond to the ^1^H NMR chemical shifts (ppm) at the center of the bin responsible for the separation. Figure reproduced from Nature Publishing Group (Clayton et al., 2006).

Irrespective of the detection technology used, multivariate statistical analysis (MVA) methods will probably be needed in order to analyse the complex spectra from a metabolic profiling experiment and to determine statistically significant differences between the spectra of, for instance, different groups of patients. These MVA methods are in two main classes: (i) unsupervised methods such as principal components analysis (PCA), where the class of the samples e.g., patients dosed with drug X or patients dosed with placebo, is unknown to the MVA algorithm and (ii) supervised methods, such as projection to latent structures (PLS), where the class of the samples is used as an input to the algorithm. In the case of supervised MVA methods, care must be exercised to avoid over-fitting of the data, and external validation of results with an independent cohort of samples is best practice. Analysis of metabolic profiling data will often start by using an unsupervised method and especially PCA to provide: (i) an overview of the spread of the samples in metabolic space, (ii) a visualization of any separation in metabolic space between subgroups of samples, and (iii) the identification of any significant outliers in the data. In PCA, the algorithm will take linear combinations of the input variables, such as NMR signal intensities, to form a series of principal components (PCs). Each successive PC will explain a decreasing amount of the variance in the data set and will be orthogonal to the preceding PCs. The PCA scores plot shows the relationships between the samples in the study across typically two or three PCs. The loadings plot shows which variables in the input data are contributing the variance observed between the samples. See Figure 1 below for an example of the use of PCA. The interested reader can find further information in the recent literature (Lindon and Nicholson, 2008; Robinette et al., 2013).

## The discovery of pharmacometabonomics in animals and humans

### The discovery of preclinical pharmacometabonomics

Irreproducibility of results was a source of concern in some early metabonomics experiments, particularly those involving metabolic profiling of animals in drug safety or drug metabolism studies. The biofluid metabolite profiles from different animals in the same group on the same study would sometimes be so strikingly different that the competence of the technicians involved in drug dosing would be questioned. Sometimes, these effects were ascribed to “biological variation,” whatever that was. In the course of a large collaboration between Pfizer Global R & D and Imperial College London, the groups of Nicholson, Everett and co-workers, particularly John Lindon, Claude Charuel and Andy Clayton, were pursuing the discovery of early biomarkers of drug toxicity. At a meeting at Pfizer Amboise, France on October 18th 2000, the topic of significantly divergent rat urine metabolite profiles was on the agenda: in one study some rats appeared to excrete very large quantities of drug-related metabolites whereas others excreted almost none. During that meeting, the then radical idea was formulated that the post-dose differences between rats in the same group were linked to differences in their pre-dose metabolic status. Experiments were designed to test these ideas in animals and then humans and they were found to hold: pharmacometabonomics was discovered (Everett et al., 2016) and is now showing promise to help deliver personalized medicine in the future.

In order to test the hypothesis that the post-dose response of an animal to drug dosing could be influenced by its pre-dose metabolic status, a series of experiments were designed and conducted, including one key experiment involving the dosing of acetaminophen (paracetamol), a widely prescribed oral analgesic, to rats. The experimental hypothesis was that the analysis of pre-dose metabolite profiles should enable a degree of prediction of the subsequent metabolism of the drug and of its toxicity (Clayton et al., 2006). Pre-dose and post-dose urine was collected from 65 rats given a single oral dose of paracetamol (600 mg/kg in an aqueous suspension): a further 10 rats were in a control group (suspension vehicle only). Following unsupervised principal component analysis of the *pre-dose* urine NMR spectra, a statistically significant association was shown between the score for principal component 2 and the *post-dose* mean liver histopathology score (*r* = −0.34, *p* = 0.007). In addition, a statistically significant but partial PCA separation between rats in liver histopathology class 1 (no or minimal necrosis) and class 3 (moderate necrosis) was confirmed with a Mann-Whitney *U*-test (*n* = 32, *p* = 0.002, Figure 1).

A statistically significant and validated model was also built using the supervised multivariate technique projection to latent structures (PLS, partial least squares), to predict the *post-dose* molar ratio of the metabolite paracetamol glucuronide to parent paracetamol (G/P) from the *pre-dose* metabolite profile. Variable influence on projection analysis showed that the most significant factor underlying this model was a positive correlation between G/P and the integral (signal area) of the region from 5.14 to 5.06 ppm in the pre-dose urine ^1^H NMR spectra, the region where the signals for ether glucuronides occur. The conclusion drawn was that the *post-dose* ratio of G/P was at least partly controlled by the ability to form ether glucuronides such as paracetamol glucuronide *prior to dosing* (Clayton et al., 2006). The publication also included preliminary pharmacometabonomics data on the prediction of toxicity in rats dosed with allyl alcohol and galactosamine hydrochloride. Pharmacometabonomics was defined as “the *prediction* of the outcome (for example, efficacy or toxicity) of a drug or xenobiotic intervention in an individual based on a mathematical model of pre-intervention metabolite signatures.” Pharmacometabonomics is defined as a prognostic or predictive methodology, in contrast to metabonomics/metabolomics which is a diagnostic methodology.

### The discovery of pharmacometabonomics in humans

**Figure 2:**
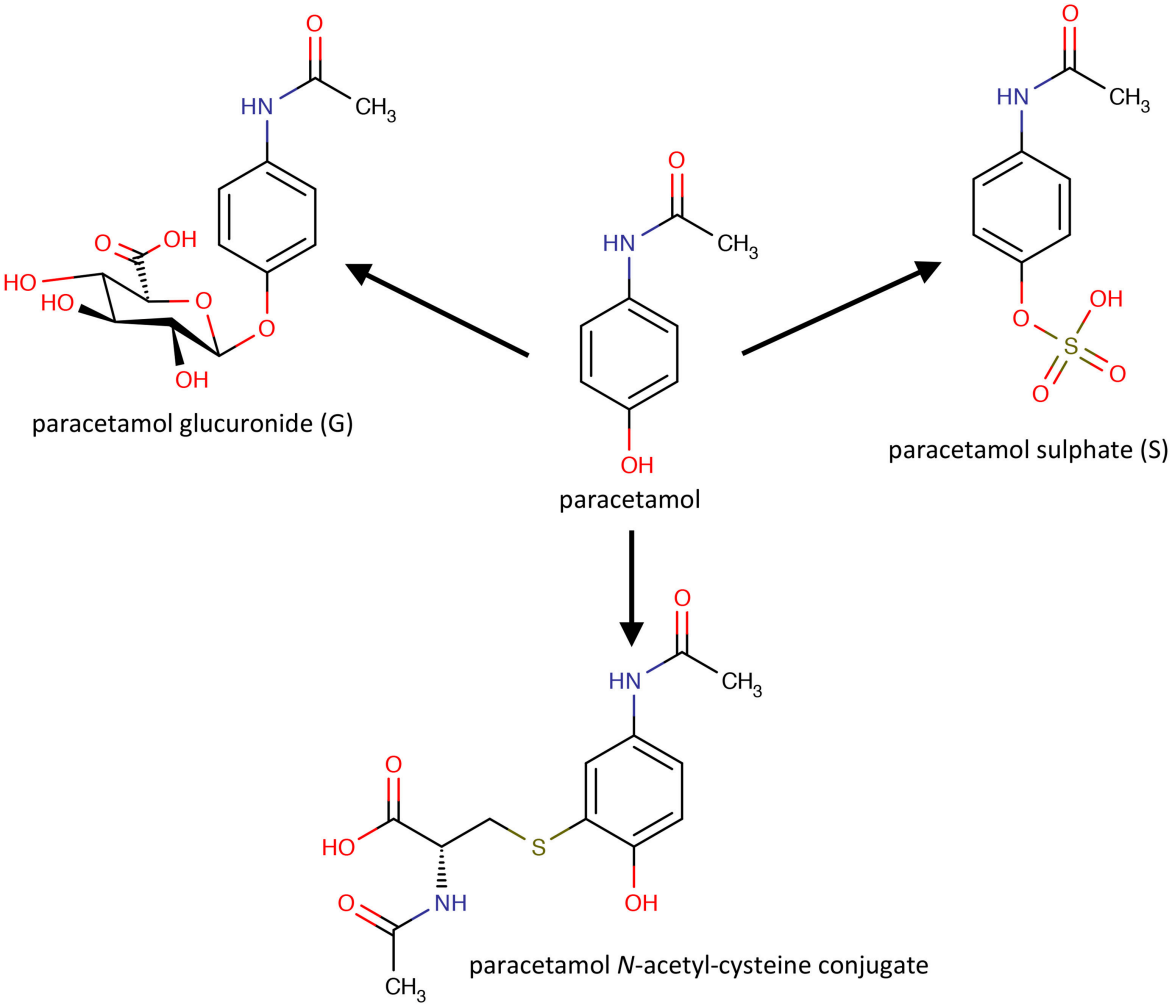
**The molecular structures of paracetamol and its principal human metabolites**.

Important as the results in rats were, the Pfizer-Imperial College team had as the ultimate goal, the demonstration of pharmacometabonomics in humans, with the aim of developing new approaches to personalized medicine. Following the positive pre-clinical results, a clinical experiment in 99 human volunteers was designed and ethically approved, involving oral dosing of paracetamol/acetaminophen. The research hypothesis of this experiment was that the pre-dose urinary endogenous metabolite profiles of the volunteers would be correlated with the post-dose differences in the metabolism of the drug. Paracetamol is a widely used analgesic that is principally metabolized to both sulfate and glucuronide metabolites in humans (Figure 2).

**Figure 3:**
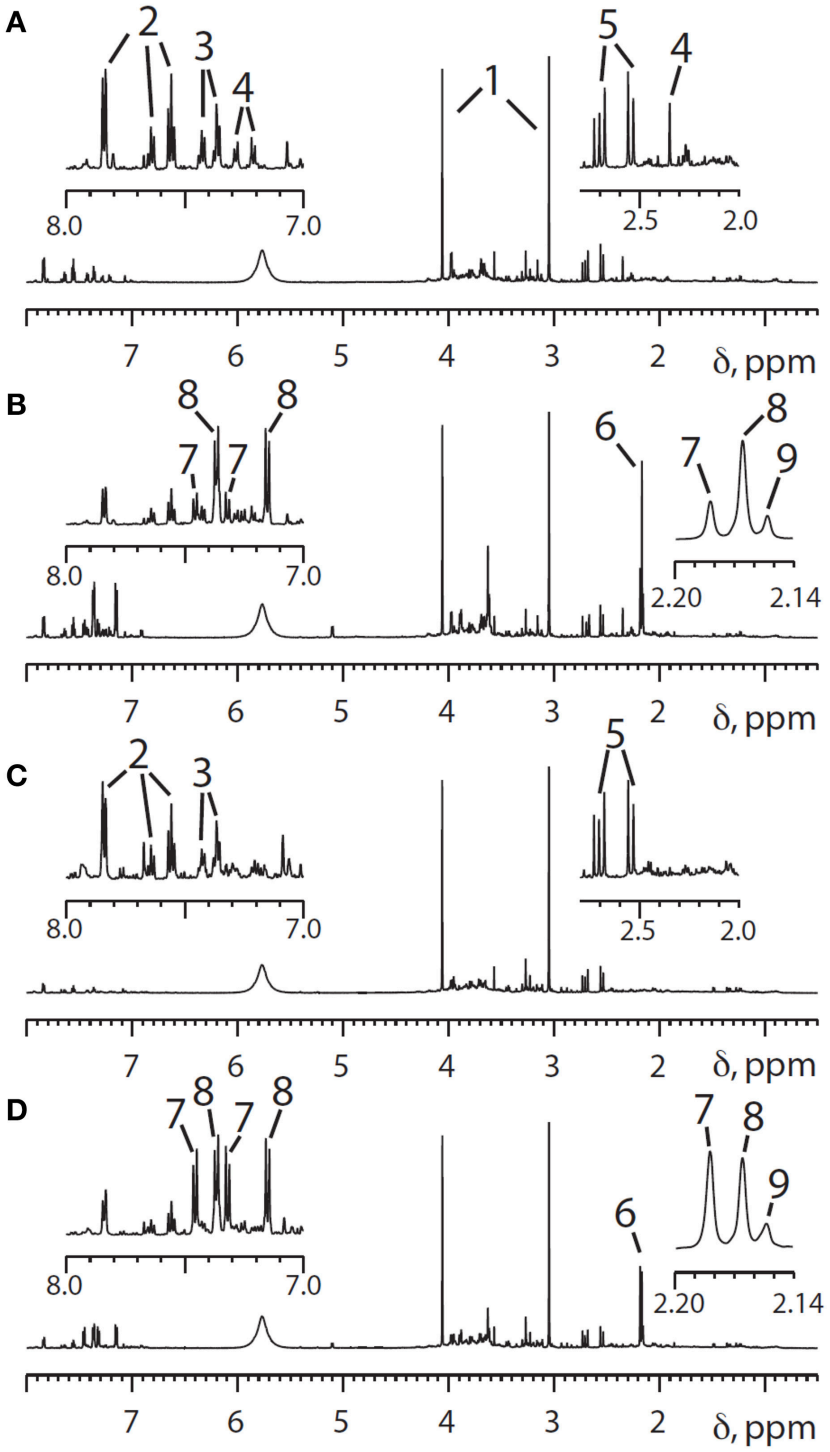
**600 MHz ^1^H NMR spectra of the urine from two different human volunteers in the region from 0.5 to 8.0 ppm, with expansion of the aromatic proton region (7.0–8.0 ppm) and a higher field region (2.0–2.8) shown above. (A)** Pre-dose urine from volunteer 1; **(B)** 0–3 h post-dose NMR spectra from volunteer 1, The main differences between the pre-dose and post-dose spectra are the appearance of signals from paracetamol and its metabolites in both the aromatic and acetyl regions. **(C,D)** Corresponding pre- and post-dose spectra for volunteer 2. Key to peak numbers: 1, creatinine; 2, hippurate; 3, phenylacetylglutamine; 4, metabolite 4 (unknown at the time: see text); 5, citrate; 6, cluster of *N*-acetyl groups from paracetamol-related compounds; 7, paracetamol sulfate; 8, paracetamol glucuronide; 9, other paracetamol-related compounds. Reproduced from PNAS (Clayton et al., 2009).

However, significant variability in the metabolism of the drug has been reported (Patel et al., 1992). Pre-dose, 0–3 and 3–6 h post-dose urines were collected from volunteers who were given a standard oral dose of paracetamol (2 × 500 mg tablets) with 250 ml water. The volunteers were not on a standard diet but were only eligible for the study if not taking drugs, herbal medicines or dietary supplements, and restrictions were placed on their diet and alcohol intake. Both the pre-dose endogenous metabolite profiles and the post-dose drug metabolite profiles were analyzed using 600 MHz flow-mode ^1^H NMR spectroscopy of the urine samples. Figure 3 shows ^1^H NMR spectra of both pre-dose and post-dose urines of two of the volunteers.

Two observations are of interest: firstly, volunteer 2 excretes a much higher ratio of paracetamol sulfate (S, metabolite 7) relative to paracetamol glucuronide (G, metabolite 8) in the 0–3 h post-dose urine and secondly volunteer 2 has no signal observable at ca 2.35 ppm in the pre-dose urine spectrum, whereas volunteer 1 has a clear and distinct signal in this region (metabolite 4).

**Figure 4:**
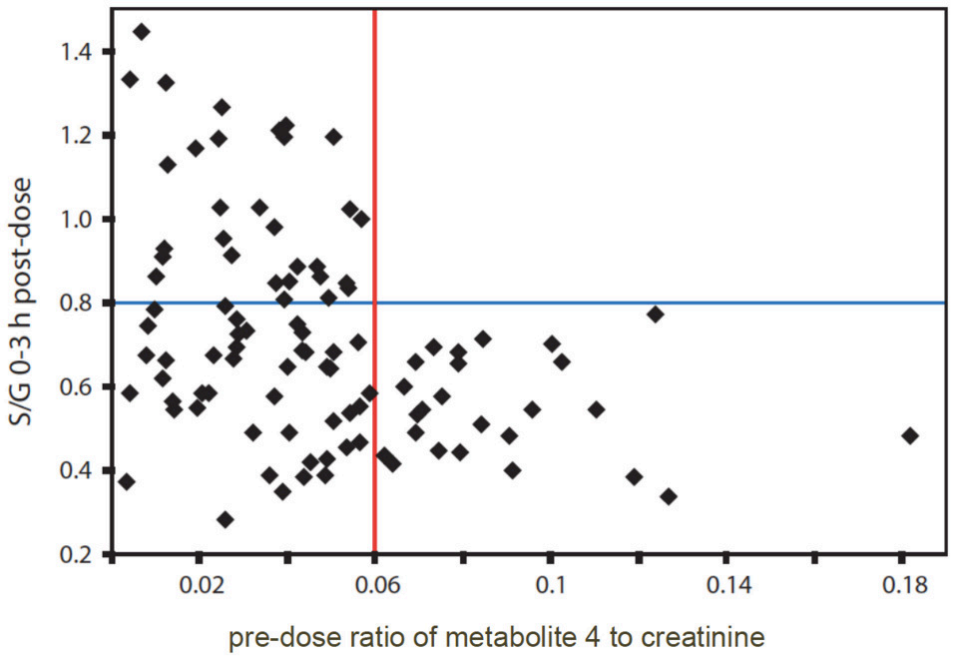
**The relationship between the 0–3 h *post-dose* ratio of paracetamol sulfate to paracetamol glucuronide (S/G) and the *pre-dose* ratio of metabolite 4, normalized to creatinine in the urines from human volunteers on the paracetamol trial**. Adapted from PNAS (Clayton et al., 2009).

Further analysis of the spectra from all remaining volunteers showed that there was a clear relationship between the presence of the signal from unknown endogenous metabolite 4 in the pre-dose urines and the ratio of S/G drug metabolites in the post-dose urines (Figure 4).

If the *pre-dose* ratio of unknown metabolite 4 normalized to creatinine was >0.06, then the *post-dose* ratio of S/G was always <0.8. However, if the pre-dose ratio of 4, normalized to creatinine was < 0.06, then the post-dose ratio of S/G took a wide range of values and was not predictable. A similar pattern was obtained after analysis of the 3–6 h post-dose urines. A Mann-Whitney test showed the statistical significance of the distribution of the high metabolite 4 to creatinine group (25 subjects) at both timepoints (*p* = 1.0 × 10^−4^ for S/G at 0–3 h and *p* = 1.2 × 10^−4^ for S/G at 3–6 h post dose). With a Bonferroni correction of 100 to mitigate for multiple hypothesis testing the *p*-value for 95% confidence is 0.05/100 = 5 × 10^−4^ (Clayton et al., 2009).

**Figure 5:**
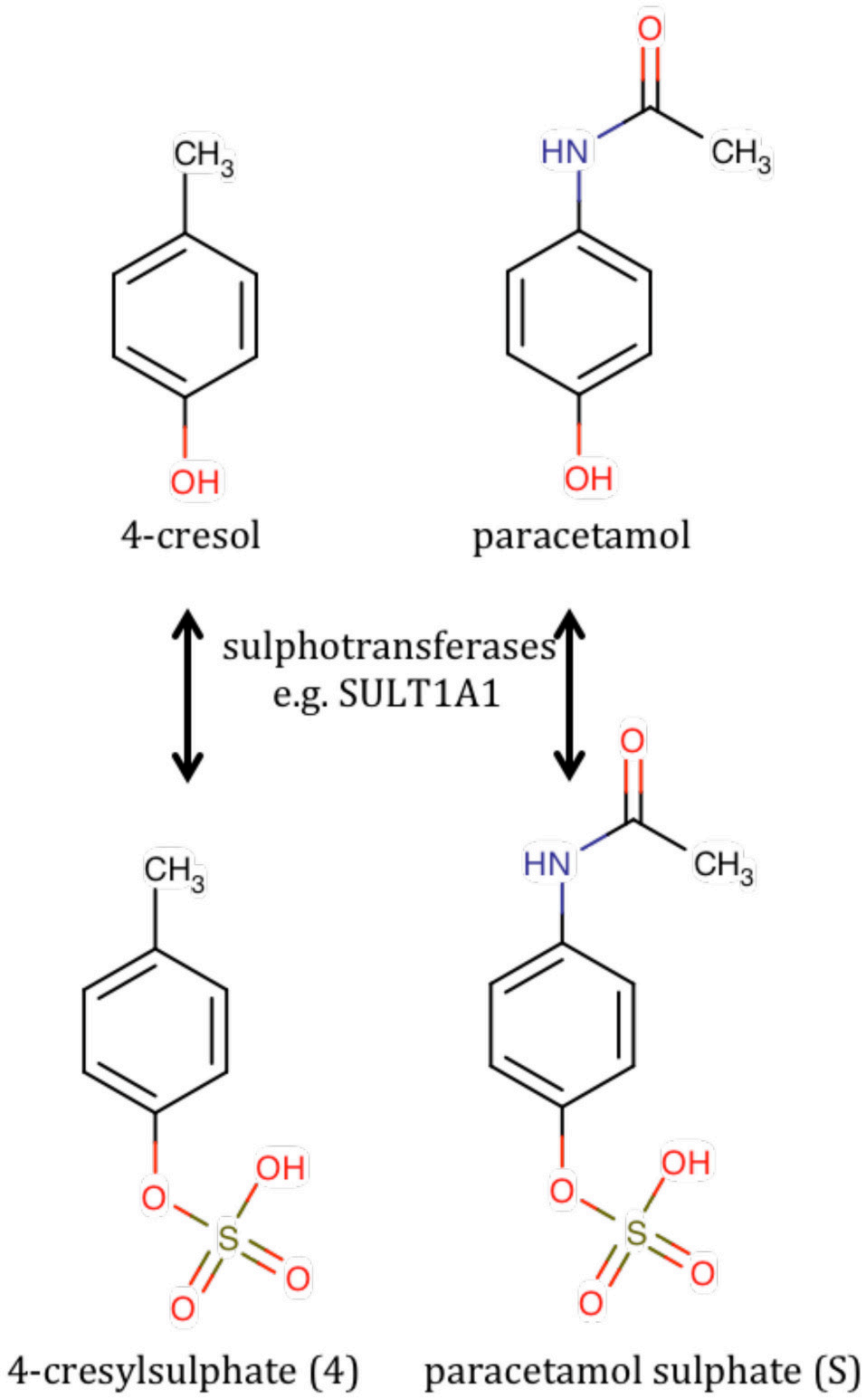
**The chemical structures of 4-cresol and its sulfation product, 4-cresylsulfate (metabolite 4) compared with the structures of paracetamol and paracetamol sulfate**.

It thus became imperative to identify unknown metabolite 4, which appeared to be a pre-dose biomarker capable of at least partial prediction of the post-dose ratio of paracetamol metabolites. Metabolite 4 exhibited a singlet resonance at ca 2.35 ppm which was assigned to a methyl group connected directly to an sp^2^ hybridized carbon atom. The singlet was associated with a pair of second-order, pseudo-doublet, aromatic proton resonances at ca 7.21 and 7.29 ppm. It was thus deduced that metabolite 4 was a 4-disubstituted toluene. Metabolite 4 was positively identified as 4-cresyl sulfate (Figure 5) by chemical, spectroscopic and enzymatic methods (Clayton et al., 2009).

The identification of the biomarker as 4-cresylsulfate came as a shock as this is not wholly a human metabolite. It is a bacterial/human co-metabolite that is produced by the human sulfation of 4-cresol, which is itself a metabolite originating in the gut microbiome, particularly from some *Chlostridia* species of bacteria (Smith and Macfarlane, 1997). In order to gain confidence in the findings, the entire NMR analysis was repeated in 2007, 4 years after the original analysis but using NMR tubes instead of a flow probe: no significant changes to the results were found. In addition, in 2008, the original NMR-based analysis of S/G for the 3–6 h post-dose urines was repeated using UPLC-MS with a correlation coefficient of 0.99 and no outliers (quantitation from online UV detector).

The rationalization of the results obtained in this paracetamol pharmacometabonomics experiment is as follows. Unlike mice and rats that can metabolites 4-cresol by sulfation or glucuronidation, humans metabolize 4-cresol largely by sulfation. In a person with a gut microbiome excreting large amounts of 4-cresol, the sulfation of this toxin to 4-cresylsulfate, metabolite 4, may use up a large part of the limited sulfation capacity of that individual. If that person is subsequently challenged with a large dose of a drug, such as paracetamol, requiring metabolism by sulfation, then the body will use glucuronidation to a greater extent instead, to make up for its diminished sulfation capacity. The situation is particularly acute for paracetamol and 4-cresol, as they will not only utilize the same sulfotransferase enzyme co-factor, 3′-phosphoadenosine 5′-phosphosulfate, which is known to be in limited supply (Coughtrie, 2002) but, due to structural similarity, they will also be in competition for the same sulfotransferase enzymes (see Figure 5; Gamage et al., 2006). Interestingly a recent study showed that germ-free mice exhibited a higher S/G ratio after dosing paracetamol than conventionally-housed mice; a result consistent with the findings in humans (Possamai et al., 2015).

The outcome of this experiment is significant (Wilson, 2009):

1. It represents the first demonstration of pharmacometabonomics in humans and provides a biological rationale for the observed inter-individual differences in drug metabolism.
2. It demonstrates that inter-individual variation in response to drug therapy is significantly influenced by the person's biochemical status and not just their genotype.
3. It shows the significant effect that the gut microbiome can have on personalized medicine for the first time.

Since many other important drugs are metabolized by sulfation, this result is expected to have significance beyond paracetamol. In addition, since sulfation is an important element of many human biochemical processes, further implications of inter-individual 4-cresol excretion from the microbiome are likely. Finally, many diseases are linked to altered levels of 4-cresylsulfate or to altered S/G ratios post paracetamol dosing, indicating that there may be a microbiome involvement in these diseases (Clayton et al., 2009).

## Progress in pharmacometabonomics in animals and humans

Since the discovery of pharmacometabonomics in animals (Clayton et al., 2006) and in humans (Clayton et al., 2009), progress in the field has been rapid. To date a total of ca 30 studies in animals and humans have been described in 36 papers (Table 1: some studies have been the subject of multiple publications). All studies up to ca 2014 have been recently reviewed (Everett et al., 2013; Everett, 2015), to which the interested reader is referred, so this review will focus on more recent developments.

**Table 1:** **The use of pharmacometabonomics to predict drug efficacy, toxicity, metabolism, and pharmacokinetics^a^**.

**Class of experiment**	**Human studies**	**Pre-clinical studies**
Prediction of pharmacokinetics (PK)	Prediction of tacrolimus PK in healthy volunteers (Phapale et al., 2010)	Prediction of pharmacokinetics of triptolide in rats (Liu et al., 2012)
	Prediction of atorvastatin pharmacokinetics in healthy volunteers (Huang et al., 2015)	
	Prediction of methotrexate clearance in patients with lymphoid malignancies (Muhrez et al., 2016)	
Prediction of drug metabolism	Prediction of metabolism of paracetamol/acetaminophen in human volunteers (Clayton et al., 2009)	Prediction of paracetamol/acetaminophen metabolism in rats (Clayton et al., 2006)
	^**^First demonstration of pharmacometabonomics in humans	^**^First demonstration of pharmacometabonomics
	Prediction of CYP3A4 induction in volunteer twins (Rahmioglu et al., 2011)	
	Prediction of CYP3A activity in healthy volunteers (Shin et al., 2013)	
Prediction of drug efficacy	Prediction of antipsychotic effects with olanzapine, risperidone and aripiprazole (Kaddurah-Daouk et al., 2007)	
	^**^First detection of metabolic efficacy markers in baseline human samples but study was small and designated hypotheses generating rather than definitive by the authors	
	Prediction of simvastatin efficacy in patients on the Cholesterol and Pharmacogenomics study (Kaddurah-Daouk et al., 2010; Trupp et al., 2012)	
	Prediction of citalopram/escitalopram response in patients with major depressive disorder (MDD; Ji et al., 2011)	
	${*}^{*}\text{First demonstration of pharmacometabonomics-informed pharmacogenomics approach to personalized medicine}$ (Abo et al., 2012) and (Gupta et al., 2016)	
	Prediction of sertraline and placebo responses in patients with MDD (Kaddurah-Daouk et al., 2011), (Kaddurah-Daouk et al., 2013), and (Zhu et al., 2013)	
	Prediction of efficacy of anti-psychotics in schizophrenia patients (Condray et al., 2011)	
	Prediction of response to aspirin in healthy volunteers (Lewis et al., 2013; Yerges-Armstrong et al., 2013; Ellero-Simatos et al., 2014)	
	Prediction of efficacy with anti-TNF therapies in rheumatoid arthritis (Kapoor et al., 2013)	
	Prediction of thiopurine-*S*-methyltransferase phenotype in Estonian volunteers (Karas-Kuželički et al., 2014)	
	Prediction of efficacy of L-carnitine therapy for patients with sepsic shock (Puskarich et al., 2015)	
	Prediction of acamprosate treatment outcomes in alcohol-dependent patients (Nam et al., 2015)	
	Prediction of blood pressure lowering in hypertensive patients treated with atenolol and hydrochlorothiazide (Rotroff et al., 2015)	
	Prediction of response in lung cancer patients (Hao et al., 2016)	
	Prediction of patient response to trastuzumab-paclitaxel neoadjuvant therapy in HER-2 positive breast cancer (Miolo et al., 2016)	
Prediction of adverse events	Prediction of weight gain in breast cancer patients undergoing chemotherapy (Keun et al., 2009)	Prediction of toxicity from paracetamol/acetaminophen dosing in rats (Clayton et al., 2006)
	^**^First demonstration of pharmacometabonomics in patients	
	Prediction of liver injury markers in patients treated with ximelagatran (Andersson et al., 2009)	Prediction of onset of diabetes in rats administered with streptozotocin (Li et al., 2007)
	Prediction of toxicity of paracetamol/acetaminophen (“early-onset pharmacometabonomics”) (Winnike et al., 2010)	Prediction of nephrotoxicity of cisplatin in rats (Kwon et al., 2011)
	Prediction of toxicity in patients with inoperable colorectal cancer treated with capecitabine (Backshall et al., 2011)	Prediction of toxicity of isoniazid in rats (Cunningham et al., 2012)
		Prediction of variability in response to galactosamine treatment in rats (Coen et al., 2012)
		Prediction of toxicity from lipopolysaccharide treatment in rats (Dai et al., 2016)

a *Significant papers are highlighted with a double asterisk with explanatory text in bold blue font*.

The majority of pre-clinical studies on animals have focused on the prediction of adverse events. One recent publication in this area by Dai et al has shown that survival and non-survival in rats treated with lipopolysaccharide can be predicted using an LC-MS- and GC-MS-based approach (Dai et al., 2016). A pre-dose serum model based on the predictive biomarkers sphingosine, sphinganine, palmitic acid, oleic acid, and cholesterol was generated and various hypotheses on the influence of inter-individual lipid metabolism differences on survival were proposed.

Far more publications have emerged recently demonstrating success with pharmacometabonomics approaches in human volunteer and patient studies.

Huang et al. used GC-MS analysis of predose plasma samples from 48 hospitalized, healthy Chinese volunteers to predict pharmacokinetic (PK) properties of atorvastatin, a lipid-lowering agent known to have significant inter-individual PK variation (Huang et al., 2015). In this study, a 15-fold variation in C_max_ and a 12-fold variation in the total drug area under the curve (AUC) were observed. Projection to latent structures (PLS) multivariate analysis methods, with both internal and external validation, were used to show that a set of 17 and 12 endogenous pre-dose plasma metabolites were able to predict C_max_ and AUC, respectively. Molecules such as 2-hydroxybutyric acid and cholesterol were found to be important components of both the C_max_ and AUC model. It was rationalized that this may be because of competition between these endogenous metabolites and atorvastatin for monocarboxylate and organic anion transporters.

Kienana et al. used similar methodology, including external validation, to predict methotrexate clearance in a group of 62 patients with lymphoid malignancies, treated with a high dose methotrexate (>1 g.m^−2^; Muhrez et al., 2016). Methotrexate is an interesting example as genetic polymorphisms in enzymes or transporter proteins involved in methotrexate clearance are known to be associated with PK variation. However, it is striking that significant course-to-course variability in methotrexate PK *in the same patients* also occurs, which means that environmental factors are also critical (Muhrez et al., 2016). A good prediction of methotrexate clearance was obtained (mean prediction error and precision for patients not in the model group were 0.4 and 21%, respectively) from a model based on 28 pre-dose urinary metabolites, including valerylglycine, 2-methylacetoacetic acid, and gentisic acid (2, 5-dihydroxybenzoic acid). Again, the model was thought to provide information on the status/ability of transporter systems in the patient pre-dose. Less good statistics were obtained for prediction of delayed elimination: 93% of normal eliminators (excellent specificity) but only 42% of the delayed eliminators were correctly predicted (poor sensitivity). Low numbers of delayed eliminators in the patient cohort prevented further model optimization.

Weinshilboum and Kaddurah-Daouk and co-workers have developed the concept of pharmacometabonomics-informed pharmacogenomics. In this approach, metabolite biomarkers found to be predictive of drug outcomes in pharmacometabonomics experiments are used as the starting point for focused pharmacogenomics experiments. The approaches used include: (i) searching for single nucleotide polymorphisms in genes related to the synthesis, transportation, and degradation of metabolites found to be discriminating biomarkers in pharmacometabonomics experiments and (ii) the use of genotype imputation (Ji et al., 2011; Abo et al., 2012). This is an important new approach to the use of pharmacometabonomics (Neavin et al., 2016). The same group has recently followed up with a pharmacometabonomics informed pharmacogenomics study on patients with major depressive disorder (MDD) in the Pharmacogenomics Research Network Antidepressant Medication Pharmacogenomics Study (PGRN-AMPS). Predose plasma serotonin levels were associated with both remission and response at both 4 and 8 weeks after initiation of MDD patient treatment with citalopram or escitalopram. Baseline serotonin concentrations were then used as a phenotype for genome-wide association studies (GWAS). The GWAS study showed a genome-wide statistically significant SNP cluster on chromosome four 5′ of *TSPAN5* and a cluster across *ERICH3* on chromosome one. Both knockdown and over-expression of these genes in a neuroblastoma cell line significantly altered the expression of four serotonin pathway genes. It was concluded that *TSPAN5* and *ERICH3* were associated with plasma serotonin concentrations and may be involved in selective serotonin reuptake inhibitor treatment efficacy (Gupta et al., 2016).

Kapoor et al. used ^1^H NMR spectroscopy to analyse pre-dose urine samples from a small number of rheumatoid arthritis patients being treated with anti-tumor necrosis factor agents (infliximab or etanercept; Kapoor et al., 2013). Both unsupervised (PCA) and supervised (PLS) multivariate analysis methods indicated that baseline urine metabolites discriminated between patients who either did, or did not, have a good response to anti-TNF therapy, according to European League Against Rheumatism criteria (sensitivity 89%, specificity 86%). Key metabolites responsible for the discrimination included histamine, glutamine, xanthurenic acid, and ethanolamine. It was proposed that the strong discriminator histamine may be a urinary biomarker for inflammation or may report on the breakdown of histidine. Repetition of the results in a larger cohort was recommended.

Karas-Kuželički et al. utilized a highly targeted and hypothesis-driven approach to investigate if *S*-adenosyl methionine (SAM) could be predictive of thiopurine-*S*-methyltransferase (TPMT) phenotype (Karas-Kuželički et al., 2014). Inter-individual dosing of 6-mercaptopurine in the treatment of childhood acute lymphoblastic leukemia is thought to be dependent upon TPMT phenotype, although measurement of TPMT pre-treatment and *TPMT* genotyping have been shown to be not as useful as hoped, with concordance between genotype and phenotype being as low as 50% in some *TPMT*-heterozygous cohorts. SAM is known to be associated with TPMT activity as it is a cofactor known to bind to the enzyme and can thus potentially influence its activity. From a set of 19 biochemical and 10 hematological parameters, and after correction for multiple hypothesis testing, only *TPMT* genotype (*p* = 1 × 10^−13^) and SAM levels (*p* = 1 × 10^−13^) were found to be significantly associated with TPMT activity in analyses of 1017 donors to the Estonian Biobank. In *TPMT* -heterozygous patients with normal TPMT activity, high SAM levels explained the disconnect between genotype and phenotype. It was concluded that in the future it would be reasonable to measure SAM levels only in *TPMT*-mutated patients. In addition, it was proposed that SAM supplementation to *TPMT*-mutated patients with low SAM levels could reduce the incidences of side effects of 6-mercaptopurine (Karas-Kuželički et al., 2014).

Inter-individual patient differences have been an issue for sepsis therapeutics. Puskarich et al. described an untargeted ^1^H NMR spectroscopy pharmacometabonomics approach to determine differences in pre-dose serum metabolite levels between survivors and non-survivors from a pilot, phase I, placebo-controlled clinical trial of L-carnitine in 31 sepsis patients (Puskarich et al., 2015). Amongst many results, baseline concentrations of 3-hydroxybutyrate (*p* < 0.001), acetoacetate (*p* < 0.001) and 3-hydroxyisovalerate (*p* < 0.001) were all significantly lower in survivors of the therapy. Low ketone body, L-carnitine-treated patients also showed a trend toward lower 1 year mortality than any other group (chi square *p* = 0.038). It has been reported that the accumulation of ketone bodies, such as 3-hydroxybutyrate and acetoacetate, may indicate impaired metabolic functioning in sepsis. It was concluded that whilst further, larger studies were needed, pharmacometabonomics approaches could help in delivering precision medicine to sepsis patients.

Alcohol use disorder (AUD) is also a complex and heterogeneous disorder with significant patient variation. Nam et al used UPLC-MS/MS analysis of patient serum in a targeted approach to determine pre-dose biomarkers that would distinguish responders (complete alcohol abstinence) from non-responders (any alcohol usage) during 12 weeks of treatment with oral acamprosate in 120 alcohol-dependent subjects (Nam et al., 2015).

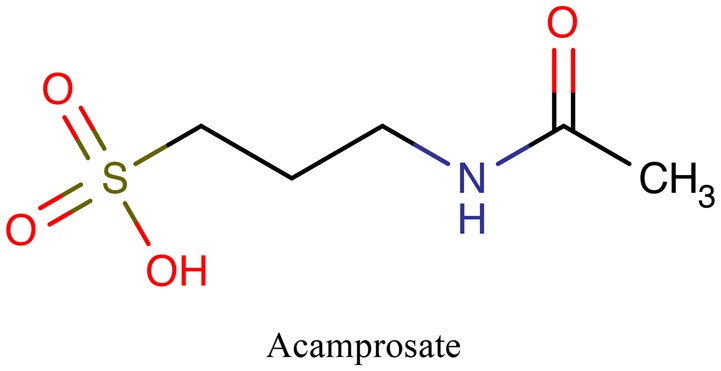



Acamprosate is known to reduce glutamate levels in the brain. This study therefore profiled 36 metabolites, including 20 amino acids and determined that pre-dose glutamate levels were significantly higher in 51 responders (32.3 ± 2.4 μM) than in the 39 non-responders (23.1 ± 1.7 μM, Wilcoxon rank sum test, *p* = 0.012) in the discovery cohort. In the replication cohort, the baseline glutamate levels were again higher in the responders (*n* = 20) than the non-responders, *n* = 10, *p* = 0.036). No impact of depression was found upon the baseline glutamate results. Serum glutamate levels were normalized in the responders after 12 weeks of therapy but were unchanged in the non-responders. Baseline serum ammonia levels were also significantly higher in the responders in the discovery cohort (*p* = 0.003) and trended higher (but not significantly) in the replication cohort. Baseline glutamate to glutamine ratios were significantly higher in the entire cohort of responders (*n* = 71) vs. non-responders (*n* = 49, *p* = 0.016). A significant drop in glu/gln ratios was seen in responders after acamprosate treatment *p* < 0.0001), whereas no effect was seen in the non-responders. Consistent with this finding, significant correlations were found between baseline glutamate and glutamine synthetase activity (*R*^2^ = 0.13, *p* < 0.05), and between baseline ammonia and glutamine synthetase activity (*R*^2^ = 0.09, *p* < 0.05) in the responders, but not the non-responders. Additional studies showed that in the absence of alcohol in mice, acamprosate enhances glutamine synthetase production of glutamine from glutamate and ammonia. It was concluded that a pharmacometabonomics approach involving measurement of pre-treatment glutamate levels could be a useful new method for personalized treatments of AUD patients with acamprosate (Nam et al., 2015).

Rotroff et al. used untargeted GC-time of flight MS to analyse plasma samples from patients enrolled in the Pharmacogenomic Evaluation of Antihypertensive Responses study at the University of Florida (Rotroff et al., 2015). One hundred and twenty-eight White and one hundred and twenty-nine black participants were randomly assigned to receive atenolol therapy whilst 123 white and 83 black participants were randomly assigned to hydrochlorothiazide therapy for hypertension. Baseline metabolite profiles were significantly different between the black and white patients (Wilcoxon rank test, *p* < 0.001), which dictated the study analysis by race. Baseline plasma levels of several metabolites, some unknown, were significantly associated with changes in home diastolic blood pressure (HDBP) in all, white and black patients on both drugs. False discovery rate was used to correct for multiple hypothesis testing with *p* < 0.05 and *q* < 0.2 considered significant i.e., the chance of the low *p*-value being a false discovery is <20%. One unknown, 223548, was positively associated with HDBP change in white patients treated with hydrochlorothiazide (*p* = 0.007, *q* = 0.15) but negatively associated in black patients (*p* = 0.004, *q* = 0.11). This is either a significant effect or possibly a false discovery. Multivariate models were built using a discovery set and a validation set (to avoid over-fitting) to predict HDBP response from baseline plasma metabolite levels. Models for all treatments/all patients; atenolol/all patients; hydrochlorothiazide/all patients and all treatments/white patients were statistically significant for both the discovery and validation sets. It was concluded that whilst additional studies were needed, pharmacometabonomics may have a significant role to play in helping deliver personalized medicine for hypertensive patients in the future.

There have also been recent developments in the use of pharmacometabonomics in the development of personalized medicine approaches for the treatment of cancer. Hao et al. have reported a pilot ^1^H NMR spectroscopy and GC-MS study of the serum of treatment-naive, non-metastatic lung cancer patients treated with standard chemotherapy, with and without radiation (Hao et al., 2016). The GC-MS analysis enabled the construction of an orthogonal PLS discriminant analysis (O-PLS-DA) model (*n* = 25, cross-validated ANOVA *R*^2^ = 0.39, *Q*^2^ = 0.29, *p* = 0.034) that showed that, amongst other differences, low pre-treatment levels of hydroxylamine, tridecan-1-ol, octadecan-1-ol were associated with survival. The GC-MS analyses provided better prognostic data, whilst the ^1^H NMR data provided better tumor diagnostic data. It was hypothesized that the biomarker alcohols are ketogenic substances and are deuterium depleted, eventually giving rise, via mitochondrial processing, to deuterium depleted “metabolic water” with a potential impact on macromolecule synthesis and cell replication (Hao et al., 2016).

Miolo et al. used an LC-MS/MS approach to identify biomarkers of response to trastuzumab-paclitaxel neoadjuvant therapy in 34 HER-2 positive breast cancer patients (Miolo et al., 2016). Histological analysis was used to categorize the patients into good responders (pathological complete response, *n* = 15) and poor responders (partial pathological response, *n* = 19). Supervised PLS-DA multivariate analysis was used to show that pre-treatment serum levels of spermidine were significantly higher in the good responders (0.15 ± 0.06 mM) vs. the poor responders [0.09 ± 0.032 mM (*p* < 0.001, *q* < 0.05)], whereas pre-treatment levels of tryptophan were significantly lower in the good responder group (61.19 ± 8.46 mM) vs. the poor responder group [73.82 ± 9.23 mM (*p* = 0.001, *q* < 0.05)]. Amongst other possibilities, it was hypothesized that high levels of spermidine could boost tumor cell replication and thus enhance paclitaxel activity, whilst low levels of typtophan may be associated with immune suppression and enhancement of tratuzumab activity (Miolo et al., 2016).

## Not pharmacometabonomics!

It is worth noting that several publications claiming to report pharmacometabonomics studies appear instead to be diagnostic metabonomics studies reporting the *effects* of drugs on metabolite profiles. These studies contain no predictive or prognostic elements and the literature is becoming confused by the mis-naming of such studies. These recent papers appear to be examples of this phenomenon (Park et al., 2013; Wikoff et al., 2013; Serrano-Contreras et al., 2016).

## Predictive metabonomics

Pharmacometabonomics is defined as “the prediction of the outcome (for example, efficacy or toxicity) of a drug or xenobiotic intervention in an individual based on a mathematical model of pre-intervention metabolite signatures.” At the time of its discovery (Clayton et al., 2006), it was already envisaged that the prediction of drug effects via pharmacometabonomics was just one example of a broader set of predictive methodologies that we now call predictive metabonomics, where the intervention could be broader than just drug treatment, for instance, diet change, physical stress, medical interventions or, just the passage of time. Predictive metabonomics has been defined simply as “the prediction of the outcome of an intervention in an individual based on a mathematical model of pre-intervention metabolite signatures” (Everett, 2015). Many excellent examples of predictive metabonomics have been demonstrated recently and some of these are directly relevant to personalized medicine as, for example, they enable the prediction of individuals who will in the future develop a particular disease vs. those who will not.

Wang et al. used LC-MS to determine that higher baseline plasma levels of leucine, isoleucine, valine, phenylalanine, and tyrosine were predictive of subjects in the Framingham Offspring Study who went on to develop diabetes over a 12 year period (*p* = 0.001 or lower). The predictive model was replicated in the Malmo Diet and Cancer Study for all amino acids except isoleucine (*p*-values 0.009–0.04; Wang et al., 2011).

Wang-Sattler et al. also used LC-MS and flow injection analysis-MS to show that lower baseline serum levels of glycine, lysophosphatidylcholine (LPC) (18:2) and acetylcarnitine were predictive of the development of impaired glucose tolerance over a 7 year period and that lower baseline serum levels of just glycine and lysophosphatidylcholine (LPC) (18:2) were predictive for the onset of type 2 diabetes over the same period (Wang-Sattler et al., 2012).

Several other examples were recently reviewed (Everett et al., 2016). In addition to these examples, Sjöberg et al. used GC-MS to demonstrate that serum myo-inositol levels, sampled 3 or 7 days after symptom onset were predictive of delayed neurological deficit as measured by the Glasgow Outcome Scale, 1 year after subarachnoid hemorrhage (Sjöberg et al., 2015).

McPhail et al. used a combined ^1^H NMR spectroscopy and UPLC-TOF-MS approach to demonstrate that levels of lysophosphatidylcholines (LPCs), phosphatidylcholines (PCs), and lipids were reduced and levels of lactate, tyrosine, methionine, and phenylalanine were increased in the plasma of 18 out of 80 decompensated liver cirrhosis (DC) patients who failed to survive 90 days post-admission (McPhail et al., 2016). The outcome from the MVA were: three component OPLS-DA (*R*^2^*X* = 0.57, *R*^2^*Y* = 0.46, *Q*^2^*Y* = 0.39) with 100% sensitivity and 85% specificity, CV-ANOVA 10^−6^. DC Patients were defined as those with an acute episode of variceal bleeding, jaundice, encephalopathy, ascites, sepsis, or renal dysfunction requiring admission to hospital. A separate cohort of 59 DC patients was used to externally validate this model: sensitivity 98% (87–100%), specificity 89% (67–99%), positive likelihood ratio 9 (3–34), negative likelihood ratio 0.03 (0.004–0.2), AUROC 0.96 (0.90–1.00), and a further cohort of 42 cirrhosis patients provided further confirmation of the 90 day mortality prediction model (McPhail et al., 2016). It was hypothesized that the lower levels of LPCs and PCs reflected increased liver damage and necrosis. Similarly, the increased levels of lactate and the amino acids were noted to reflect liver damage and failure. The fact that the model performed so well in double external validation is very encouraging and it is hoped that this new approach may have clinical utility in the short term.

## Genome-wide association studies (GWAS)

A number of important studies have been published establishing linkages between genetic variation, metabolic phenotype and disease risk or drug effect variation. Using a UPLC-MS and GC-MS approach, Suhre et al. identified 37 genetic loci associated with serum metabolite concentrations in participants from the German KORA F4 (*n* = 1768) and British TwinsUK (*n* = 1.052) studies (Suhre et al., 2011a). Another study by Suhre et al using a ^1^H NMR approach in a different cohort reported five new loci with very significant associations (*p*-values < 3 × 10^−19^) with metabolite concentrations (genetically determined metabotypes, GDMs) with three of the loci previously associated with clinical outcomes, including *SLC7A9* for chronic kidney disease, *NAT2*, for coronary artery disease and *SLC6A20* for iminoglycinuria (Suhre et al., 2011b).

A large study by Kettunen et al identified 31 genetic loci with significant associations with human serum ^1^H NMR-based metabolite levels in a GWAS of 8330 Finns genotyped and imputed at 7.7 million SNPs (Kettunen et al., 2012). The heritability of the metabolite measures was assessed using intra-pair metabolite correlations for 221 monozygotic and 340 dizygotic twins from the Finnish Twin Cohort and was estimated as varying between 23 and 55% for amino acids and other small molecules, 48–62% for lipids and 50–76% for lipoproteins. Examples of key loci identified include *SLC25A1* with citrate, *DHDPSL* with glutamine and *SLC1A4* with valine.

Rhee et al. reported a GWAS of 217 MS-detected, human plasma metabolites in 2076 participants of the Framingham Heart Study. Estimated heritability was found to explain >20% of inter-individual variation (Rhee et al., 2013). A total of 23 previously unidentified genetic loci associated with plasma metabolites were discovered.

Shin et al. reported a large GWAS reporting significant genome-wide, associations between 145 loci and >400 metabolites, using GC-MS/MS and LC-MS/MS analysis of serum and plasma from 7824 adults from the KORA and TwinsUK data sets (Shin et al., 2014). The study comprehensively linked the resulting data with information on gene expression, heritability, and overlap with previously identified genetic loci for diseases, inborn errors of metabolism and pharmacological targets. A powerful network model of genetic and metabolic associations was built. The contribution of metabolic loci to variance in metabolite concentrations was in the range 1–62% with a median value of 6.9%.

Rueedi et al. described a GWAS linking genetic variation with human urine ^1^H NMR metabolite features in 835 Caucasians from the *CoLaus* study (Rueedi et al., 2014). Out of 139 discovered associations, 56 replicated and the identity of the underlying metabolites was sought from the NMR features using a new metabomatching method. In this way, SNP rs281408 in *FUT2* was significantly associated with fucose concentrations and a linkage with Crohn's disease established.

It is clear that GWAS studies such as these, and other related studies (Nicholson et al., 2011) will have a significant role to play in the understanding of the origin of inter-individual variance in metabolite concentrations and on the relative roles of genetic and environmental factors to such variation. These studies will undoubtedly help reach the goal of personalization of medicine in the future.

## Conclusions

Metabonomics is a young, diagnostic science that was defined < 2 decades ago (Lindon et al., 2000) but it has already spawned a new prognostic methodology, pharmacometabonomics, defined as: “the prediction of the outcome (for example, efficacy or toxicity) of a drug or xenobiotic intervention in an individual based on a mathematical model of pre-intervention metabolite signatures” (Clayton et al., 2006), that has shown great promise in predicting the efficacy, safety, metabolism, and pharmacokinetics of drugs prior to dosing. Most of the studies published to date are relatively small pilot studies, but larger, externally validated studies are emerging more recently: the study by McPhail et al. demonstrating external validation in two independent cohorts of a model for the prediction of survival in decompensated liver cirrhosis patients is a landmark (McPhail et al., 2016).

Pharmacometabonomics is expected to work well together with pharmacogenomics and the landmark pharmacometabonomics-informed pharmacogenomics study of Abo et al. demonstrated the power of these two technologies working together in concert (Abo et al., 2012). The development of many genome wide associations between genetic variation, metabolic phenotype and disease risk, or drug response variability is an important development in this area and is expected to help interpret the results of both pharmacogenomics and pharmacometabonomics experiments.

It is exciting to see the emergence of the new field of predictive metabonomics, where the effects of interventions broader than just drug treatment can be predicted. Remarkable predictions of disease onset have been achieved, sometimes predicting events that occur years after the baseline metabolic profile measurements.

Another exciting development is that of longitudinal pharmacometabonomics, where the journey of a patient through a clinical episode can be tracked and predictions of best treatment options made (Nicholson et al., 2012).

The development and coordinated use of all these methods is poised to impact upon the improved delivery of personalized medicine for patients, as pharmacogenomics on its own is likely to be insufficient to fully deliver the promise of optimized treatment for specific sub-groups of patients. It is envisaged that pharmacometabonomics in concert with pharmacogenomics and metagenomic analysis of patient microbiomes will provide the wealth of relevant data necessary to make critical personalized decisions for patient healthcare. That future is now close to us.

## Author contributions

JE conceived and wrote the review article.

### Conflict of interest statement

JE is a co-inventor on a recently granted patent on pharmacometabonomics (EP1540560 and WO 03/107270 A2).

## Footnotes

^1^The concept of metabonomics arose in 1996 in the course of a collaboration between Jeremy Nicholson at Birkbeck College, University of London and Jeremy Everett at Pfizer Global R & D, in order to provide a framework for the biofluid NMR spectroscopy elements of a series of what would now be called multi-omics or pan-omics studies using genomics, proteomics, clinical chemistry, and biofluid analyses in an integrated fashion to discover novel, early, drug safety biomarkers. Metabonomics was chosen as the etymologically-preferred term.

## Abbreviations

^1^H: hydrogen-1
GC: gas chromatography
GDM: genetically determined metabotype
GWAS: genome-wide association studies
HPLC: high performance liquid chromatography
JRES: J-resolved
LC: liquid chromatography
MS: mass spectrometry
MVA: multivariate (statistical) analysis
NMR: nuclear magnetic resonance
PCA: principal components analysis
PK: pharmacokinetics
PLS: projection to latent structures (partial least squares)
SNP: single nucleotide polymorphism
TOF: time-of-flight
UPLC: ultra performance liquid chromatography.
